# T Lymphocyte Migration: An Action Movie Starring the Actin and Associated Actors

**DOI:** 10.3389/fimmu.2015.00586

**Published:** 2015-11-18

**Authors:** Loïc Dupré, Raïssa Houmadi, Catherine Tang, Javier Rey-Barroso

**Affiliations:** ^1^INSERM, UMR 1043, Centre de Physiopathologie de Toulouse Purpan, Toulouse, France; ^2^Université Toulouse III Paul-Sabatier, Toulouse, France; ^3^CNRS, UMR 5282, Toulouse, France; ^4^Master BIOTIN, Université Montpellier I, Montpellier, France

**Keywords:** T lymphocytes, migration, actin cytoskeleton

## Abstract

The actin cytoskeleton is composed of a dynamic filament meshwork that builds the architecture of the cell to sustain its fundamental properties. This physical structure is characterized by a continuous remodeling, which allows cells to accomplish complex motility steps such as directed migration, crossing of biological barriers, and interaction with other cells. T lymphocytes excel in these motility steps to ensure their immune surveillance duties. In particular, actin cytoskeleton remodeling is a key to facilitate the journey of T lymphocytes through distinct tissue environments and to tune their stop and go behavior during the scanning of antigen-presenting cells. The molecular mechanisms controlling actin cytoskeleton remodeling during T lymphocyte motility have been only partially unraveled, since the function of many actin regulators has not yet been assessed in these cells. Our review aims to integrate the current knowledge into a comprehensive picture of how the actin cytoskeleton drives T lymphocyte migration. We will present the molecular actors that control actin cytoskeleton remodeling, as well as their role in the different T lymphocyte motile steps. We will also highlight which challenges remain to be addressed experimentally and which approaches appear promising to tackle them.

## Introduction

Cell motility relies on the remodeling of cell shape, a process which is highly controlled both in time and space to allow cell polarization and coordination of movement in response to extracellular cues (1). The integration of motility signals into coordinated cell shape remodeling is ensured by the actin cytoskeleton. The building block of the actin cytoskeleton is the globular actin, which polymerizes into filament networks of various complexity depending on the levels of actin filament branching and crosslinking (2). Actin networks that undergo rapid and dynamic remodeling provide the physical basis for the emission of diverse types of protrusions that allow complex motility tasks such as environment probing, cell body translation, and invasion through natural tissue barriers. *Via* its anchorage to cell surface receptors allowing attachment to the cell substratum and its association with molecular motor proteins such as myosins, the actin cytoskeleton sustains mechano-sensing and mechano-transduction, allowing the cell to both sense the physical constraints of its environment and assemble force generating protrusions that ultimately lead to cell body translation (3).

In most migrating cells, the front or leading edge is made of a thin and widely spread structure called the lamellipodium. It is composed of highly branched actin fibers that compose a dense meshwork. The lamellipodium undergoes periodical contractions that are coupled to a retrograde actin flow (4). The cell leading edge is also characterized by the presence of thin longilineal protrusions of various lengths called filopodia that carry out an exploratory function (5). Parallel bundles of cross-linked actin fibers are the structural basis for filopodia. These protrusions can either be embedded within the lamellipodium or be emitted independently from it. Cells such as immune cells and tumor cells that have the capacity to cross tissue barriers made of dense extracellular matrix (ECM) networks assemble invadopodia or related structures that can locally digest the ECM to allow cell invasion. In some cells such as lymphocytes, the leading edge structure can be a pseudopodium, which is a more bulky protrusion than the lamellipodium since it is filled with cytoplasm. Behind the cell leading edge, the shape of the cell body is maintained by the actin cortex, a thin network of actin fibers localized beneath the cell membrane. Alternative motility strategies, not depending on the assembly of a lamellipodium, can be ensured by the formation of membrane blebs as a result of hydrostatic pressure from within the cell and local relaxation of cortical actin (6). The cell rear or trailing edge is generally characterized by actin filament bundles coupled to myosin. This allows the sliding of actin fibers that generate cell tension driving the cell body and cell rear forward. Importantly, each cell type is endowed with specific motility characteristics, which are reflected by different abilities to remodel the actin cytoskeleton to support the assembly of protrusions.

In this context, lymphocytes are classified as cells exhibiting amoeboid motility. Indeed, their motility characteristics are comparable to those described in the *Dictyostelium discoideum* amoeba. The morphology of migrating lymphocytes is characterized by the emission of actin-rich pseudopodia, blebs, and a highly contractile trailing edge referred to as the uropod. The amoeboid motility of lymphocytes [reviewed in Ref. (7)] is further characterized by weak adhesion to the substratum and little or no ECM proteolysis. The motility of lymphocytes is intimately related to their function as immune sentinels and effectors. Indeed, lymphocytes can migrate extremely rapidly, adapt their motility strategies to cross different tissue barriers, and orient their migration in response to various chemotactic factors. In addition, the motility behavior of lymphocytes tunes the quality of their interaction with antigen-presenting cells (APC). How the specific features of lymphocyte migration are controlled by the underlying actin cytoskeleton is only partially elucidated. The objective of this review is to cover the current knowledge on how specific molecular aspects of actin cytoskeleton remodeling contribute to T lymphocyte motility characteristics. We also aim at pointing to the unsolved questions and to the approaches that could help unraveling them.

## Actin Cytoskeleton Dynamics in Migrating T Lymphocytes

### Overview of T Lymphocyte Motility

T lymphocytes are among the cells displaying the highest exploratory behavior. They are able to migrate all around the body and to explore most tissues. Beyond those large-scale displacements, T lymphocytes continuously scan their local environment in a manner favoring recognition of specific antigens at the surface of APC. Motility is therefore an inherent property of T lymphocytes that guarantees optimal and timely recognition of foreign antigens as well as local activity at the site of the antigenic challenge. The development of intravital two-photon microscopy has permitted the investigation of T lymphocyte motility directly in tissues (8). This motility can be assimilated to a search behavior that has been characterized as a Brownian random walk (9) or a Lévy walk (10). T lymphocyte motility is known to be driven by local factors such as the architecture of the stromal network (11) and chemokines (12). The encounter with APC modifies the dynamic behavior of the T lymphocytes since the recognition of the cognate antigen by the TCR halts migration (13–15). Even if many environmental cues modulate T lymphocyte motility, many T lymphocyte motility features appear to be cell intrinsic. Indeed, the speed as well as the stop and go behavior of T lymphocytes is comparable under *in vivo* and *in vitro* conditions lacking stromal network and chemokines (16). In addition, T cells autonomously regulate their ability to turn while migrating, a key parameter for the efficiency of the meandering search of rare antigens (17).

### T Lymphocyte Motility Parameters

To describe and understand more precisely the motility behaviors of T lymphocytes, it is crucial to define parameters and to verify that they are measured appropriately (18). In this chapter, we will provide an overview of the parameters pertaining to speed, direction, and cell shape that are being used to characterize and compare motility patterns of T lymphocytes both *in vitro* and *in vivo*.

Although T lymphocytes are very rapid cells, their speed is far from being constant. To measure the speed of lymphocytes is not a straightforward task since not all considered lymphocytes might migrate during the window of observation, and because a key feature of T lymphocyte motility is the alternation of running and pausing phases. A first basic point is therefore to consider the proportion of actively migrating lymphocytes within a population of interest. It has been long established that upon isolation from human blood, only a portion of lymphocytes display spontaneously a motile behavior (19). Transwell assays performed to measure the ability of T lymphocytes to migrate toward a chemokine consistently show that not all cells migrate, even if they express apparently homogeneous levels of the corresponding chemokine receptor. What distinguishes the motile versus non-motile fractions of lymphocytes is currently unknown. However, the activation state might be related to the propensity to migrate, since lymphocyte stimulation with mitogens can result in a majority of lymphocytes to acquire a motility behavior (20).

To appreciate the alternation of running and pausing phases, it is important to image T lymphocytes over sufficient periods of time. In 3D collagen lattices, T lymphocytes display periods of highly directional migration alternating with frequent turns (21). Under these conditions, the mean velocity of human unstimulated lymphocytes is approximately 7 μm/min (excluding non-migrating cells and periods of pausing), with half of the cells migrating. Following stimulation with Concanavalin A, the proportion of migrating cells increased to 80% without any change in the velocity. Can T lymphocyte velocity be tuned? In addition to the well-established role of chemokines in driving directional migration, chemokine might exert a chemokinetic role. During CD8^+^ T cell infiltration of the murine brain in the context of Toxoplasma infection, CXCL10 increased the velocity of T lymphocytes and their ability to find infected target cells, without modifying the walk pattern (10). T lymphocyte velocity also seems to be increased during (dendritic cell) DC scanning (22). Indeed, T lymphocytes migrate within the paracortex of lymph nodes at a high speed (10–15 μm/min on average) (23, 24). In comparison, naive T lymphocyte locomotion in the subcapsular region was reported to be 38% slower and had higher turning angles and arrest coefficients than naive T lymphocytes in the deep paracortex (25). Is T lymphocyte velocity dependent on their phenotype? Memory T lymphocytes were shown to move faster during CCL21-driven chemokinesis when compared to naive T lymphocytes (26). The higher motility characteristics of memory T lymphocytes were associated with an increased propensity to attach to the substratum. In addition, T regulatory cells exhibit higher cell motility properties (basal and across barriers) than the non-T regulatory cell counterparts (27).

The random walk trajectories that T lymphocytes follow are influenced both by the architecture of the reticular network and by interactions between cells. This is particularly true during scanning of lymphoid organs, as T lymphocyte motility is halted upon TCR-dependent antigen recognition at the surface of APC (28). Indeed, a key aspect of the motility of T lymphocytes is the stop and go behavior directed by the encounter with APC and in particular DC (24). The parameter usually used to assess this behavior is the arrest coefficient, which corresponds to the proportion of time in which a T cell does not move (threshold <2 μm). The arrest coefficient is generally low when T cells are not engaged in stable contacts with DC, with a baseline mean arrest coefficient value around 0.35 (29). In the presence of antigen, the mean arrest coefficient is increased to 0.5 and above. Interestingly, T regulatory cell inhibitory activity can be mediated through preventing the arrest of effector T cells. Indeed, in a murine model of experimental autoimmune encephalomyelitis, the absence of T regulatory cells significantly decreased the speed and increased arrest (0.70) of specific CD4^+^ T cells in the presence of auto-antigen (30).

The exploratory behavior of T lymphocytes is usually appreciated by calculating the confinement ratio, which is defined as the ratio of the distance between the initial and the final positions of a cell to the total distance covered by that cell. In a murine model of subcutaneous thymoma, the exploratory behavior of cytotoxic T lymphocytes was more restrained in the antigen-bearing tumors (confinement ratio of 0.4), compared with tumor that did not present specific antigens (confinement ratio of 0.6) (31). This parameter also revealed that cytotoxic T lymphocytes resumed motility following tumor cell killing. This shows that motility parameters evolve as a T cell response is developing.

Another aspect useful to appreciate T lymphocyte motility is the study of the turnings. A couple of studies focusing on *in vitro* T cell tracking have reported that periods of turning coincide with a slower motility (26, 32). The assessment of turnings via an angle analysis is useful to assess whether cells tend to be attracted toward preferential positions or cellular partners. This has helped to show that naive CD8^+^ T cells are attracted to sites of CD4^+^ T cell–dendritic cell interactions (33). Additionally, the measurement of the angle between the directions of travel of T cells as a function of the distance between each other revealed the presence of dynamic microstreams of naive T cells in lymph nodes (34).

The shape of T lymphocytes is also an important parameter to assess their motility behavior. As we will see in details below, the shape of T lymphocytes is constantly remodeled as they migrate due to a combination of protrusive and contractile activities. In addition, cell shape can be informative about the physical interaction of T lymphocytes with tissue structures. For example, during tumor infiltration, cytotoxic T lymphocytes were shown to frequently move along blood vessels. In doing so, they adopted a particularly elongated morphology, which was assessed by calculating an elongation index (ratio between the length and the width of the cell) (31).

### Actin Cytoskeleton and Shape Remodeling Driving T Lymphocyte Migration

As for any motile cell, actin cytoskeleton remodeling plays a central role in T lymphocyte motility (35). Beyond the classical rules of cell motility mediated by a dynamical actin cytoskeleton, we will consider here what is specific about T lymphocytes. A migrating T lymphocyte harbors a highly dynamical leading edge, a contractile central region and an adhesive uropod. It undergoes periodical leading edge extension, central region traction, and uropod retraction. It ensues an amoeba-like motility behavior (36). As observed for *D. discoideum* amoebas, lymphocytes migrate in a cyclic fashion characterized by peaks of high velocity interrupted by pausing periods (37). Velocity peaks correlate with pseudopod extension and pauses correlate with both pseudopod contraction and generally rounder cell morphology (32).

**Figure 1:**
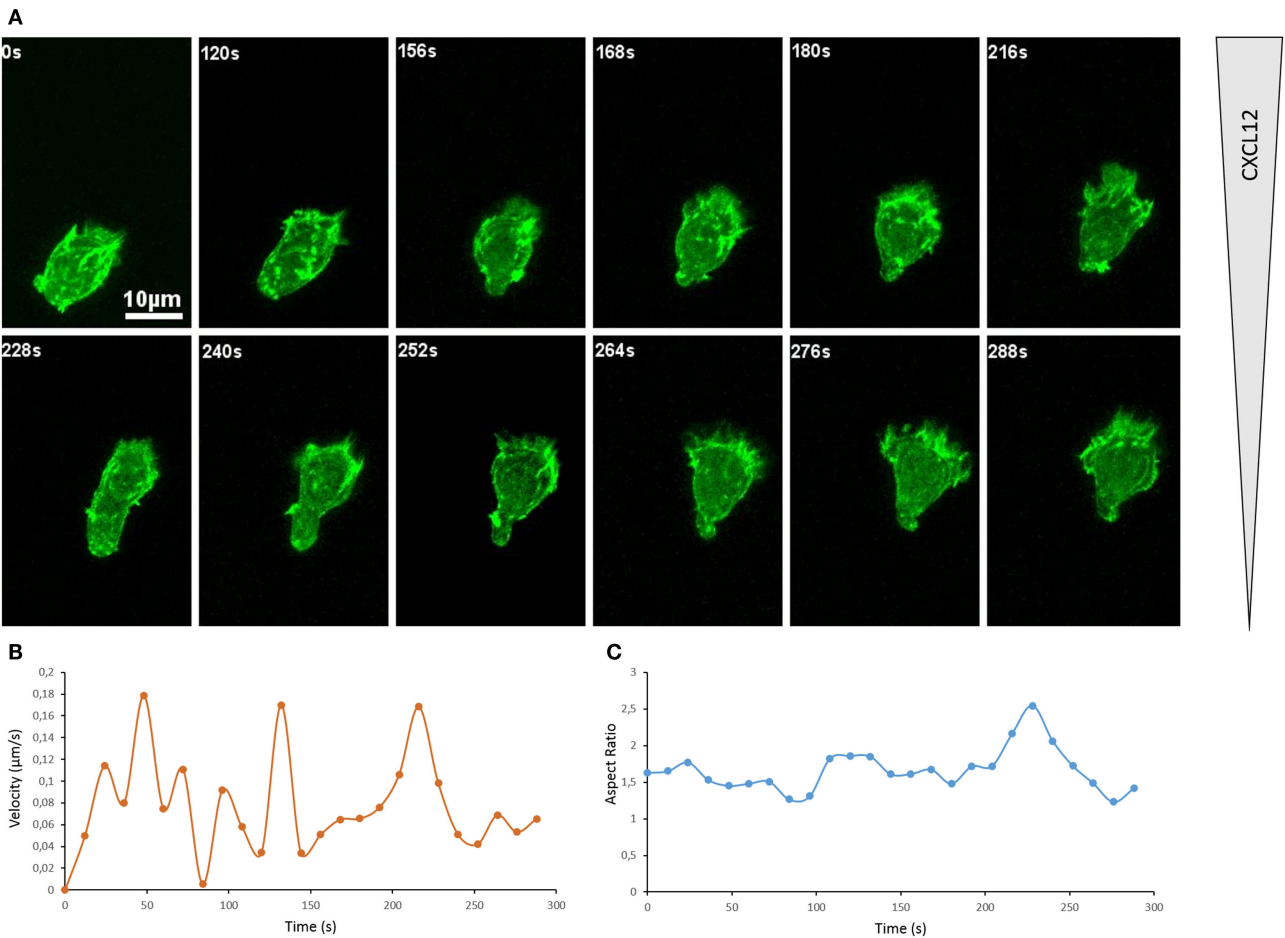
**Actin cytoskeleton underlies T cell morphological changes during directional migration**. **(A)** Snapshots of a movie showing a primary CD8^+^ human T cell expressing Dendra2-LifeAct moving along a CXCL12 gradient created in a collagen IV-coated Ibidi μ-Slide Chemotaxis^2D^. The cell extends dynamic protrusions and moves toward the source of CXCL12 (top). See Movie S1 in Supplementary Material. The organization of the actin cytoskeleton in T lymphocytes can also be appreciated in previous reports on the dynamics of actin during scanning of target cells (40) and polarization in response to CXCL12 (41). **(B)** Velocity of the cell shown in **(A)**, calculated for successive 12 s intervals on the basis of the tracking of the cell. **(C)** Morphology of the cell shown in **(A)**, calculated as Aspect Ratio (length/width of a fitted ellipsis) for each frame.

A widely used approach to visualize actin cytoskeleton dynamics in living cells is to use a fluorescently tagged actin or fluorescently tagged LifeAct, a short peptide that binds polymerized actin filaments (38). With the scope to image the actin cytoskeleton of a primary T lymphocyte, we have electroporated Dendra2-LifeAct in CD8^+^ T lymphocytes purified from human peripheral blood. These cells were then exposed to a CXCL12 gradient generated in a chemotaxis microchamber. As depicted in Figure 1A, at most of the snapshots, the cell is clearly polarized pointing toward the source of CXCL12 (see also Movie S1 in Supplementary Material). The leading edge is enriched in actin and is under constant remodeling. Intense ruffling occurs at this site. Consequently, an unstable actin-rich structure is assembled, which takes the shape of a pseudopodium for most of the time. When T lymphocytes are studied on 2D surfaces like it is the case on these images, the leading edge might also transiently adopt a structure approaching that of a lamellipodium (time frames 264 and 276 s). In the case of our short movie, while the primary T lymphocyte is constantly remodeling its shape, it manages to move along a very directional path. When plotting the displacement of the centroid of the cell (Movie S1 in Supplementary Material), we appreciate that the cell is moving toward higher concentration of CXCL12 (top) with some oscillations of direction. A useful way to measure the directionality of a cell along a linear gradient of known orientation is to calculate the forward migration index, which is defined as the ratio between the cell displacement length along the gradient axis and the track length (39). This calculation yields a value of +0.76 for the lymphocyte depicted here, which indicates a relatively straight and directed path. For theoretical comparison, a cell that would migrate along a perfect line perpendicularly to the gradient would yield a value of +1, while a cell that would move but not progress along the gradient axis would yield a value of 0. Interestingly, when measured over 12 s intervals, the velocity of the primary lymphocyte along its path is highly variable (Figure 1B). Indeed, it varies from 0.01 to 0.35 μm/s. Over the 288 s of the registration, the T lymphocyte migrates at a mean velocity of 0.16 μm/s or 9.6 μm/min. This is compatible with velocities recorded by two-photon microscopy in tissues. Roughly, it corresponds to the estimation that a lymphocyte migrates at a speed of one body length per minute. In parallel, the general morphology of the cell is changing as well, as it can be appreciated by plotting the Aspect Ratio (length/width of a fitted ellipsis) over time (Figure 1C).

**Figure 2:**
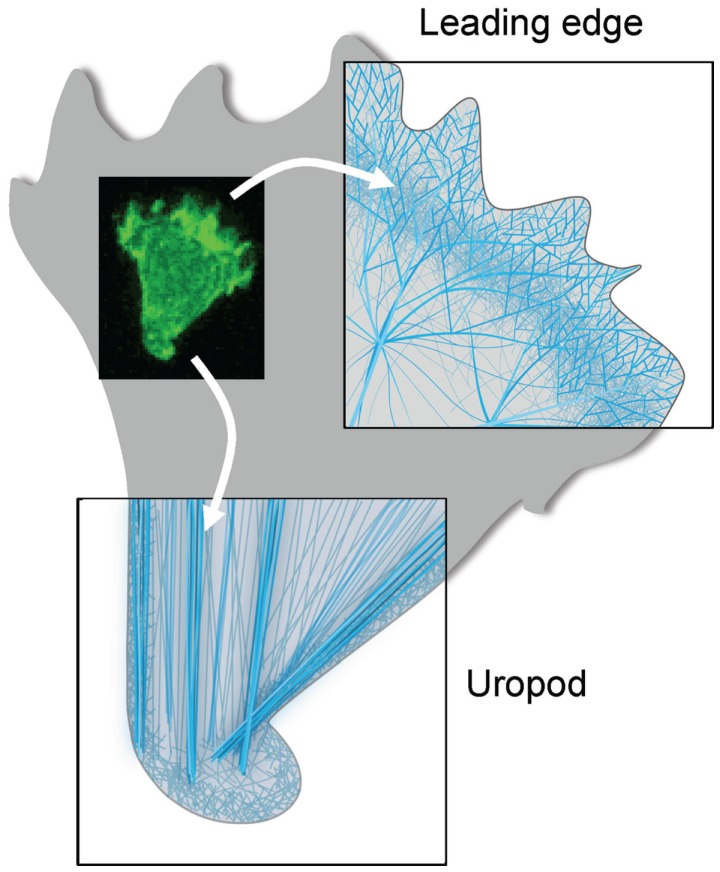
**Actin cytoskeleton organization at the two poles of a migrating T cell**. Schematic representations of the ultrastructure of the actin cytoskeleton networks at the leading and trailing edges of the migrating T cell shown in Figure 1. At the leading edge, the T cell that migrates on a 2D surface emits a protrusion that alternates between a lamellipodium and a pseudopodium. It contains a very dynamical and highly branched actin meshwork. At the trailing edge, the T cell uropod is made of a network of parallel actin bundles that can slide along each other to generate contractile forces.

Let us now consider more in details the type of actin remodeling at work in the lymphocyte as it migrates directionally. For that purpose, we show in Figure 2 a schematic representation of the organization of the actin cytoskeleton filaments both at the leading edge and the uropod of the lymphocyte studied in Figure 1. This representation shall only be considered as a model since the ultrastructure of the actin cytoskeleton network has not yet been characterized in details in lymphocytes. At the leading edge, the polymerization and branching of actin filaments provide an engine for cell movement that pushes forward the cell membrane. The represented structure is inspired from that of a lamellipodium structured by a branched actin meshwork. At the trailing edge, the T cell uropod is structured by a network of parallel actin bundles that that serve as a basis for contractile forces. Note that a thin layer of actin, called the cortical actin, coats the inner side of the plasma membrane. This actin pool is important for cell shape maintenance and changes. The rest of the cell probably contains a 3D network of crosslinked filaments interspersed with contractile bundles.

The simplified setting employed here to visualize actin cytoskeleton and cell shape remodeling in primary T lymphocytes illustrates the inherent nature of motility in these cells. Beyond the dynamic structures described above, it is important to stress that motile T lymphocytes can assemble additional actin-rich structures depending on the stimulatory context. Indeed, those cells have developed distinct migratory strategies, in particular as they need to cross the endothelial barriers, as they migrate through dense tissue environments and as they search for antigens.

**Figure 3:**
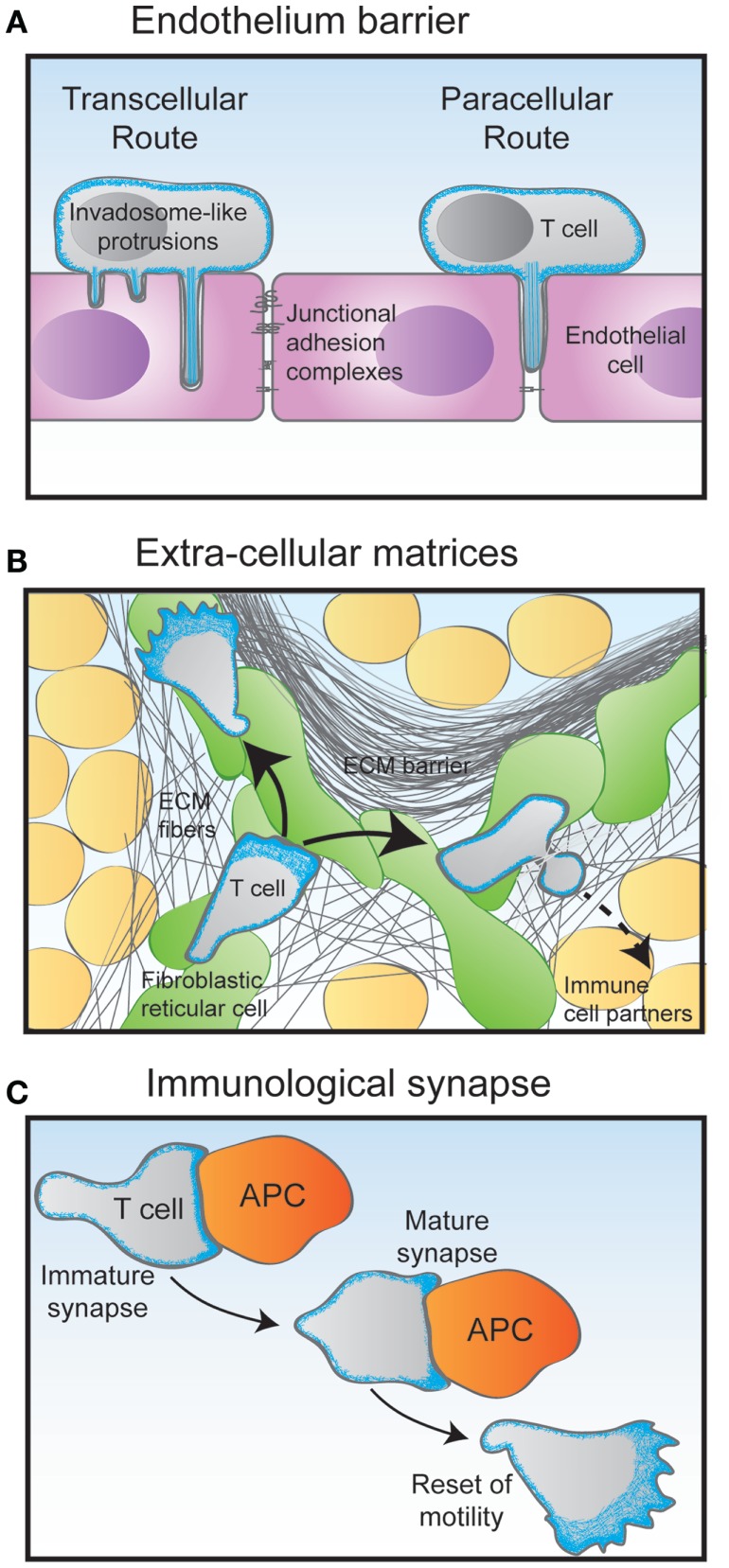
**Migratory challenges faced by T cells during their journey through the organism**. **(A)** The crossing of the endothelium barrier follows steps of T cell tethering and rolling on the luminal surface of the endothelial cells. The combination of chemokines and adhesion molecules triggers firm adhesion to the endothelial surface via the emission of filopodia-like protrusions. Depending on their activation state, T cells then either use the transcellular route by emitting invadopodia-like protrusions that go across the entire endothelial cell body, or the paracellular by squeezing through the junction between two adjacent endothelial cells. **(B)** Following the crossing of the endothelial barrier and underlying basal membrane, T cells undergo interstitial migration through the tissue they have entered in. Using an amoeboid mode of motility, they crawl and squeeze along and through extracellular matrix (ECM) fibers of various nature. In the lymph node cortex, T cells preferentially migrate along a network of fibroblastic reticular cells decorated with chemokines. **(C)** During the scanning of antigen-presenting cells (APC), T cells make multiple encounters of various duration and quality. Some contacts may last only a few minutes in the form of an immature immunological synapse. Upon recognition of an APC bearing specific antigens, the T cell stops migrating to assemble a long-lasting immunological synapse. In addition to controlling the interaction between the T cell and the APC, the dynamical actin cytoskeleton serves as a physical platform for numerous signaling events that take place at the immunological synapse to activate the T cell. After a few hours, the activated T cell detaches from its APC partner and regains its motility behavior.

The control of T lymphocyte motility is a key to the multistep process of extravasation from the blood stream into tissues. The mechanisms of tethering, rolling, firm adhesion, and transendothelial migration have been well described (42). Interestingly, upon interaction with endothelial cells, T lymphocytes crawl with an amoeboid motility. The shear stress exerted by flowing blood is contributing to the formation of LFA-1-dependent adhesions (43). T lymphocytes orient their migration against fluid flow as they interact with the inner surface of blood vessels (44). A recent study suggests that upstream flow mechanotaxis may only rely on a passive self-steering mechanism, whereby the uropod would serve as a microscopic wind vane (45). In particular, lymphocytes have been shown to extend dynamic protrusions during transendothelial migration (Figure 3A). These actin-rich exploratory structures have been characterized as either podosomes or filopodia, depending on the lymphocyte activation state (43, 46). They appear to allow lymphocytes to scan endothelial cells and to identify areas favorable for transcellular diapedesis. The nature of the endothelium may also determine different requirements from the T lymphocyte side. This is revealed by the role of uropod contractility in transendothelial migration to reach the lymph node but not the bone marrow (47).

The scanning behavior of T lymphocytes is evident as they migrate through both lymphoid and peripheral tissues. In these complex 3D environments, packed with multiple cells types embedded in ECM structures of different composition, porosity, and elasticity, T lymphocytes appear to not require functional integrins to migration (48). Instead, they use the force of actin-network expansion, which promotes protrusion of the leading edge. Contractions delivered by the actomyosin network allow the squeezing of the T lymphocyte through narrow gaps (Figure 3B).

Another key event in the T lymphocyte life cycle is the repeated encounters with antigen-presenting cells (Figure 3C). The immunological synapses formed at the interface between T lymphocytes and APC are crucial sites for setting the duration, strength, and quality of the antigen stimulation. These dynamic structures can form in the context of both a relative arrest of migration and an ongoing scanning of the APC surface (49). They rely on actin cytoskeleton remodeling for initial assembly as well as for controlling dynamical molecular events (50, 51). Indeed, via a centripetal flow, the actin cytoskeleton provides a framework for signaling cluster movement (52). A newly added facet of actin cytoskeleton remodeling at the immunological synapse is the assembly of actin dots at the site of TCR engagement. Such dots are formed *de novo* as a result of TCR engagement and allow amplification of distal signaling events required for optimal T lymphocyte activation (53). The most detailed view of actin remodeling at the T cell synapse has been provided recently by the use of 4D super-resolution microscopy (40). The use of lattice light-sheet microscopy allowed to image dynamic actin in 3D at a high speed. What it unravels is that lamellipodial membrane protrusions form the initial contact with the target cell. Within approximately 1 min, the contact site flattens and actin gets depleted from the center of the contact area. This remodeling is associated with a rearward flow of actin filaments. Such remodeling allows MTOC docking and lytic granule delivery in the case of cytotoxic T lymphocytes.

## Molecular Control of Actin Cytoskeleton Remodeling in T Lymphocytes

We have seen above that actin cytoskeleton remodeling is an essential component of the numerous dynamic processes involved in T lymphocyte motility. We will now review the molecular machinery that allows for actin remodeling, with a focus on the molecules whose function has been elucidated in T lymphocytes. For clarity, Table 1 provides a list of the molecules described throughout the text and depicted in the figures.

**Table 1:** **Molecular actors contributing to actin cytoskeleton remodeling in T lymphocytes**.

Protein	Gene	Function	Associated immune-related disease	Reference
a-actinin	*ACTN1*	Actin crosslinking	ACTN1-related thrombocytopenia	(54, 55)
Arp2	*ACTR2*	Actin branching	n.r.	(2, 50)
Arp3	*ACTR3*	Actin branching	n.r.	(2, 50)
Cathepsin X	*CTSZ*	Uropod detatchment	n.r.	(56, 57)
Cdc42	*CDC42*	Filopodia assembly	n.r.	(58)
CIP4	*TRIP10*	Membrane curvature, WASP activation	n.r.	(59–61)
Cofilin	*CFL1*	Filament dissasembly	n.r.	(62)
Coronin1A	*CORO1A*	Arp 2/3 inhibition	Moderate to severe combined immunodeficiency	(63–65)
DOCK2	*DOCK2*	Rac1 activation	Primary immunodeficiency with early-onset invasive infections	(66, 67)
DOCK8	*DOCK8*	Cdc42 and Rac1 activation	Primary immunodeficiency with impaired cellular and humoral immunity	(68–70)
Drebrin	*DBN1*	Actin bundling, microtubule interaction	n.r.	(71–73)
Ezrin	*EZR*	Actin-transmembrane proteins crosslinking	n.r.	(74–76)
Filamin A	*FLNA*	Actin crosslinking	n.r.	(77–79)
Formin-like 1	*FMNL1*	Filament elongation	n.r.	(80)
Gelsolin	*GSN*	Filament capping	n.r.	(81)
HS1	*YWHAQ*	Branching stabilization	n.r.	(82–84)
INF2	*INF2*	Filament elongation	n.r.	(85)
Kindlin3	*FERMT3*	Actin-integrins interplay	Autosomal recessive leukocyte adhesion deficiency syndrome-III (LAD-III)	(86)
L-plastin	*LCP1*	Actin bundling	n.r.	(41)
mDIA1	*DIAPH1*	Filament elongation	n.r.	(87–89)
MLCK	*MYLK*	Actomyosin contraction	n.r.	(90)
Moesin	*MSN*	Actin-transmembrane proteins crosslinking	n.r.	(76)
Mst1	*STK4*	Chemokine receptor-integrins interplay	T cell immunodeficiency	(91–93)
Myo1g	*MYO1G*	Actin contraction	n.r.	(17)
MyoIIA	*MYH9*	Actomyosin contraction	n.r.	(90, 94–98)
Paxillin	*PXN*	Actin-integrins interplay	n.r.	(99–101)
PI3K	^a^	PI(3,4,5)P3 generation	n.r.	(66, 102, 103)
Profilin	*PFN2*	Actin polymerization	n.r.	(104, 105)
Rac1	*RAC1*	Actin branching (lamellipodia)	n.r.	(58)
Rap1	*RAP1A*	Chemokine receptor-integrins interplay	n.r.	(106–110)
RapL	*RASSF5*	Chemokine receptor-integrins interplay	n.r.	(91, 108)
RhoA	*RHOA*	Actomyosin contraction	n.r.	(111)
Rock	*ROCK1*	Actomyosin contraction	n.r.	(90)
Sharpin	*SHARPIN*	Actin-integrins interplay	n.r.	(112)
srGAP2	*SRGAP2*	membrane curvature, mDia1 inhibition	n.r.	(113)
Talin	*TLN1*	Actin-integrins interplay	n.r.	(114)
Vinculin	*VCL*	Actin-integrins interplay	n.r.	(115)
WASH	*WASH1*	Arp 2/3 activation	n.r.	(116)
WASP	*WAS*	Arp 2/3 activation	Wiskott–Aldrich syndrome	(53, 117–119)
WAVE2	*WASF2*	Arp 2/3 activation	n.r.	(115, 120, 121)
WIP	*WIPF1*	WASP activation	Primary immunodeficiency resembling the Wiskott–Aldrich syndrome	(122–124)

*n.r., not reported*.

*^a^For a list of the many genes encoding the various PI3K subunits and isoforms, see Ref. (102)*.

### Basic Regulation of Actin Asssembly

**Figure 4:**
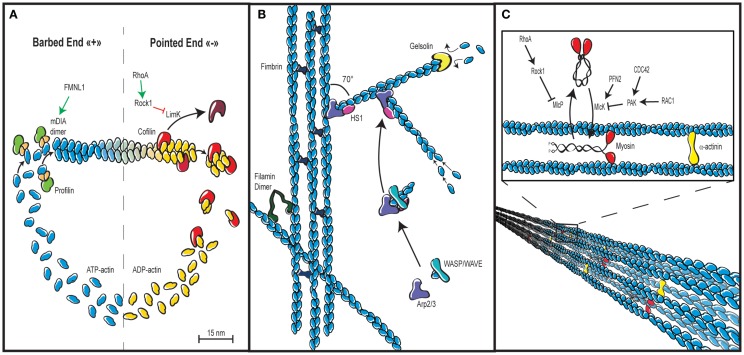
**Actin cytoskeleton architecture**. **(A)** Actin cytoskeleton dynamics rely in part on the tightly controlled cycle of polymerization and depolymerization, also known as treadmilling. ATP-bound actin is added to the fast growing barbed end of filaments via the combined action of profilin, which prevents self-nucleation of actin monomers and actin-nucleating proteins such as the formin FMLN1 or WASP-family proteins, both of which are under the control of RhoGTPases. Depolymerization is promoted by cofilin, which stimulates dissociation of ADP-bound actin at the pointed end of filaments. The rate of cofilin-mediated depolymerization can be controlled by Rho via Rock and LimK. **(B)** In addition to be elongated by formins, actin filaments can build networks in multiple ways. Actin bundles or cables with parallel or anti-parallel orientation of actin filaments are assembled by cross-linking proteins such as fimbrin. Actin filaments can also be cross-linked in a non-parallel fashion via filamin to create a gelled network. Branched networks are promoted by the Arp2/3 complex that initiates nucleation of branched filaments on the side of existing ones. This activity is driven by WASP-family proteins and stabilized by HS-1. An additional important regulation of actin cytoskeleton networks is mediated by capping proteins such as gelsolin, which bind the plus end of actin filaments to prevent monomer exchange. **(C)** Actin filaments not only generate forces while they elongate. They also generate the cell contractile forces via the intercalation of the molecular motor myosin between parallel actin filaments, which results in filament sliding. Such process is regulated by the control of the myosin light chain phosphatase and kinase activities, as well as by the degree of actin cross-linking via α-actinin.

The thermodynamics of actin filament assembly and turnover have been extensively studied as recently reviewed in Ref. (2). The building block is the actin monomer, which can form dimers and trimers. If a critical local concentration of free ATP-bound actin monomers is available, a double-stranded helical and polar filament is assembled (Figure 4A). The two ends of an actin filament are distinct. The barbed end (plus end) is more dynamic than the pointed end (minus end) since it elongates 10 times faster. The spontaneous assembly of actin filaments is kept under control by profilin, which inhibits the formation of actin oligomers. The function of this essential protein has not specifically been addressed in the context of T lymphocyte biology. Actin filaments tend to be capped by capping proteins, which stabilizes actin filament length by preventing both actin monomer association and dissociation at the filament barbed end. In T lymphocytes, over-expression of the capping protein gelsolin inhibits their spreading on plate-bound anti-CD3 antibodies (81). However, the role of gelsolin in the context of T lymphocyte motility has not been investigated so far. A requirement to further enhance actin polymerization is the uncapping or severing of the capped actin filaments. Cofilin is severing actin at the pointed end to provide free barbed ends that can serve as a template for further actin polymerization (Figure 4A). Under conditions not favorable to polymerization, the action of cofilin will on the opposite lead to a depolymerization of filaments. In T lymphocytes, the modulation of cofilin activity has been shown to occur downstream of costimulatory molecules (62).

### Actin Filament Elongation, Branching and Stabilization

The build-up of high-order actin cytoskeleton networks relies on actin-binding proteins that promote and organize actin nucleation and therefore filament assembly. Such proteins are actin nucleation factors such as formins and Arp2/3, as well as upstream promoting factors such as the WASP and WAVE family proteins.

Formins are actin nucleators that interact with the barbed end of actin filaments to further elongate them (Figure 4A). This activity leads to the elaboration of an unbranched actin network. Formins studied so far in T lymphocytes include mDIA1, FMNL1, and INF2. Via its actin polymerization activity, mDia1 is involved in T cell chemotaxis as well as TCR-driven proliferation (87–89). Interestingly, formins are also important regulators of microtubule cytoskeleton orientation in T cells, such as during the formation of the immunological synapse (80, 85, 125). By the way, an interesting area of research will be to unravel the dynamic connections between the actin and tubulin cytoskeletons, which are most probably involved in key steps of T lymphocyte motility and activation.

Alternatively to the assembly of novel filaments or the elongation of existing ones, polymerization can take a different form. Indeed, the Arp2/3 complex branches new actin filaments to pre-existing filaments with an angle of 70° (Figure 4B). This activity is crucial in that it leads to the elaboration of a branched actin network. The activation of Arp2/3 is under the control of the WASP family and the WAVE family.

WASP is a well-studied actin regulator in hematopoietic cells, including T lymphocytes, because its deficiency is the cause of the Wiskott–Aldrich syndrome, a rare primary immunodeficiency. The lack of WASP results in abnormalities of T cell homing *in vivo* (126). Whether this is the consequence of reduced chemotaxis (117), transendothelial migration (46), or interstitial migration still needs to be clarified. In addition, WASP appears as a key regulator of immunological synapse stability (118, 119) and signaling by bridging both LFA-1 (16) and the TCR (53) to the actin cytoskeleton. WASH is a WASP family member that regulates Arp2/3-dependent endosomal trafficking processes. In T lymphocytes, it controls the recycling to the membrane of key receptors including the TCR, CD28, LFA-1, and GLUT1 (116). How its deficiency impacts on T lymphocyte migration remains to be studied.

WAVE2 is the main WAVE family member expressed in T lymphocytes. It is activated by Rac1 downstream the TCR as a multi-protein complex. It is required for the spreading of the lamellipodium, conjugate formation, actin accumulation at the immunological synapse, and Ca^2+^ entry from the extracellular medium (120). Upon TCR stimulation, WAVE2 interacts with vinculin and talin (activities of which are defined in the Section “Actin-Integrins Interplay”), thereby promoting the inside-out signaling that leads to the conformational activation of integrins (115, 121). Such activities place WAVE2 as a prominent actin regulator of the mobility and activation of T lymphocytes.

The WASP-interacting protein (WIP) was initially described as a chaperone protein for WASP. However, T lymphocyte defects are more pronounced in WIP-deficient patients and mice as compared to their WASP-deficient counterparts. Indeed, WIP deficiency is associated with a disruption of the actin cytoskeleton network and defective chemotaxis (122, 123). This can be explained by the fact that WIP stabilizes actin filaments independently of its binding to WASP. This property of WIP was recently found to be critical for the integrity of the actin cytoskeleton in T cells and for their migration into tissues (124).

HS1 is the hematopoietic homolog of cortactin. It binds WASP, promotes weakly the activity of the Arp2/3 complex, and stabilizes branched actin filaments (Figure 4B). In the absence of HS1, T lymphocytes display unstable lamellipodia, as well as reduced Ca^2+^ influx and TCR-driven proliferation (82–84). The exact role of HS1 in the regulation of cytoskeleton dynamics in the context of T cell polarity and motility remains to be elucidated.

Another regulator of the Arp2/3 complex expressed in T lymphocytes is Coronin-1A (63–65). Mutations in the encoding gene are the cause of a primary immunodeficiency. Coronin-1A associates with and inhibits the Arp2/3 complex. As a homotrimer, it binds both the actin and the plasma membrane to link extracellular signals to the actin cytoskeleton. It is required to control the actin cytoskeleton remodeling driving T lymphocyte trafficking, in particular, the egress from the thymus.

### Actin Filament Crosslinking and Myosin-Driven Contraction

A pleiotropic cross-liker of actin filaments is the protein α-actinin (Figure 4C). In lymphocytes, it was initially reported to control receptor aggregation (54). Interestingly, in migrating T lymphocytes, it bridges intermediate-affinity LFA-1 to the actin cytoskeleton at the leading edge. This activity controls the attachment of the leading edge, and therefore, both cell spreading and migration (55).

Filamins are dimeric actin-binding proteins that differently from α-actinin, crosslink actin filaments in a non-parallel fashion (Figure 4B). They also play an important role as scaffolding proteins since they link intracellular signaling molecules to the actin cytoskeleton and to cell membrane receptors. In T lymphocytes, filamin A stabilizes the inactive/low affinity conformation of integrins and mediates the outside-in signaling via a cross-linking to the actin cytoskeleton. Consequently, filamin A regulates T lymphocyte motility (77). At the immunological synapse, filamin A colocalizes with PKCθ to favor TCR/CD28-driven activation (78). It also mediates the costimulatory signal of CD28 by permitting its recruitment to lipid rafts (79).

Another level of actin filament assembly is controlled by actin-bundling proteins that favor the association of multiple actin filaments in a parallel fashion, resulting in the formation of cable-like structures. T lymphocytes express the actin-bundling protein L-plastin, which localizes to the leading edge in response to CXCR4 stimulation and regulates polarity and migration (41). Interestingly, T-plastin expression in lymphocytes is restricted to malignant lymphocytes from Sézary syndrome patients, in which it regulates chemotaxis (127). This observation suggests that abnormal T lymphocytes may hijack actin cytoskeleton regulators to acquire specific motility properties.

As highlighted above, the dynamic interconnections between the actin and tubulin cytoskeletons are probably key events in the organization and tuning of lymphocyte polarity and mobility. Drebrin is an actin-microtubule coupling protein that possesses a cryptic actin bundling activity. It colocalizes with the chemokine receptor CXCR4 and F-actin at the periphery of the immunological synapse (71). It also plays a role in the regulation of extracellular calcium influx (72). In the context of HIV infection, it acts as a negative regulator of virus entry and virus-mediated cell fusion (73).

As mentioned in the previous chapters, the association of actin bundles with the molecular motor protein myosin produces contractile forces that are essential to regulate leading edge dynamics, cell body contractions, and uropod retraction. Myosins belong to very large family that has not yet been very much investigated in hematopoietic cells (128). They form heteromeric complexes by the association of heavy and light chains. Myosins form anti-symmetrical mini-filaments that, once incorporated within an actin network, provoke actin filament gliding, and thus local contraction and tension. The activity of the class II myosin MyoIIA (or Mhc9) in T lymphocyte migration is controlled by integrin and chemokine receptor-induced signals. Active forms of the upstream regulators Myosin Light Chain Kinase (MLCK) and ROCK are also present during lymphocyte migration. These kinases phosphorylate myosin regulatory light chains at distinct locations: MLCK is active at the leading edge to control adhesion and extension of the lamellipodium, while ROCK is involved in the retraction of the uropod (90). During the crossing of the endothelial barrier, MyoIIA facilitates T lymphocyte migration by squeezing the rigid nucleus through the endothelial junctions (94). T lymphocytes can rapidly switch between an adhesion-dependent sliding motility and an amoeboid walking motility that depends on MyoIIA (95). In addition to the density of integrin ligand, T lymphocytes can adapt their motility mode to the degree of environmental confinement (96). The immunological synapse is the site of an actin retrograde flow combined with actomyosin II contractions. Those contractions are generated by concentric actomyosin II arcs/rings at the periphery of the immunological synapse (97). These highly dynamic events are involved in the inward movement of TCR microclusters, a key process in the integration of antigenic signals by the T lymphocyte. T lymphocytes also express the class I myosin Myo1g. In a recent study, murine T lymphocytes genetically deleted for Myo1g were reported to have global reduction in membrane tension (17). However, their homeostatic tissue distribution and responsiveness to TCR stimulation were normal. The abnormality of Myo1g-deficient T lymphocytes resides in the fact that they moved faster and straighter. As a consequence, these cells scanned their environment too quickly for an optimal detection of rare antigens.

## Actin Cytoskeleton Interaction with Receptors Driving T Lymphocyte Migration

**Figure 5:**
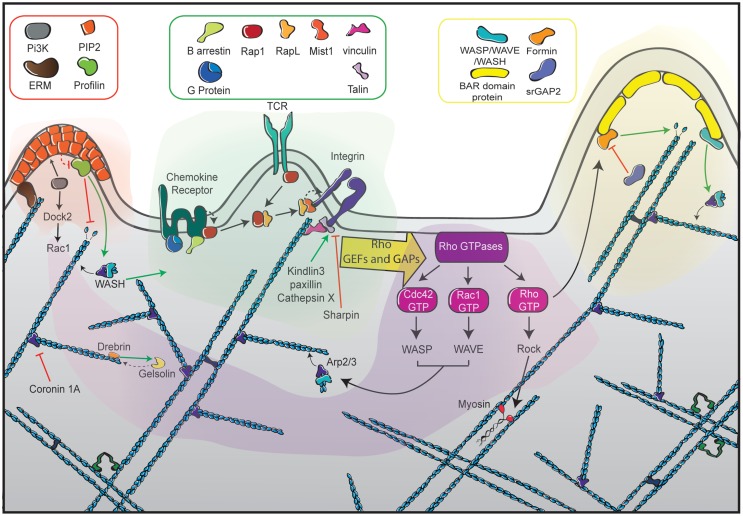
**The different facets of actin cytoskeleton remodeling in migrating T cells**. Represented at the center of the scheme (green zone) are the dominant receptors in the control of T cell motility. They include chemokine receptors, the TCR and integrins such as LFA-1, each being interconnected with the actin cytoskeleton with specific sets of signaling molecules. Ligand-mediated triggering of these receptors leads to the activation of the RhoGTPases Rac, Rho, and Cdc42 via GEFs and GAPs (purple zone). Such activation is highly controlled in time and space to orchestrate the assembly of distinct actin networks. Rac activates the WAVE complex, leading to Arp2/3-mediated actin polymerization at the leading edge to form a branched actin network. Cdc42 also favors membrane extension by activating the Arp2/3 complex via WASP. In addition to its major role at the uropod, Rho plays a dual role at the leading edge by promoting actin filament elongation via the formin mDia and by favoring membrane retraction via myosin activation. The left side of the scheme depicts the role of ERM proteins as anchors of the actin cytoskeleton in the plasma membrane (orange zone). The right side of the scheme illustrates the role of BAR-domain proteins as molecular links to guarantee local coordination of membrane curvature and actin polymerization (yellow zone). The actin meshwork is represented as blue filaments that are intertwined with the different signaling areas.

As anticipated in the previous chapters, the multiple facets of actin cytoskeleton remodeling that drive T lymphocyte migration need to be highly coordinated in time and space in response to extracellular cues. As illustrated in Figure 5, we will discuss herein the privileged relationships that the actin cytoskeleton maintains with the main surface receptors controlling T lymphocyte motility: integrins, chemokine receptors, and the TCR.

### Actin-Integrins Interplay

The integrin repertoire expressed by T lymphocytes and the molecular activation of integrins are tightly regulated phenomena that dictate the homing of these cells. In migrating T lymphocytes, the anchorage of integrins to the actin cytoskeleton is going to give a “fulcrum” to exert traction forces. Indeed, mature adhesions serve as an anchor to exert pulling forces through the contraction of actin bundles. The connection the actin cytoskeleton is also key in regulating the assembly and disassembly of integrin mediated adhesions. The interaction between integrins and the actin cytoskeleton is not direct and requires the participation of several proteins.

In the nascent adhesions associated with the leading edge of migrating lymphocytes, the action of talin is considered key to drive integrin activation (129). Upon chemokine-dependent signaling, the leukocyte specific talin-1 binds to LFA-1 β2 cytoplasmic tail (114) in a PIP2-dependent way (130), inducing integrin switch to an open conformation of high affinity. Ligand binding and talin connection with the cortical actin will stabilize this high-affinity conformation and strengthen the cell–matrix adhesion (114). This integrin–actin molecular bridge will be reinforced by kindlin-3 binding to the integrin β chain (86) and regulated by multiple cytoplasmic interactors such as the adhesion scaffold protein paxillin. Paxillin associates to the intracytoplasmic tail of β-integrin and interacts with many of its regulators such as vinculin. Paxillin activity controls adhesion assembly and turnover, thereby driving LFA-1-dependent T lymphocyte spreading and motility (99, 100). In the context of T lymphocyte migration across the endothelium, paxillin is involved in VLA-4 adhesiveness to low density VCAM-1 under shear stress conditions (101).

Vinculin is a physical interactor linking the cytoplasmic tail of integrins to the actin cytoskeleton. In leukocytes, it has been mainly studied in the context of immunological synapse assembly, where it is recruited by the Cdc42 effector WAVE2 (115, 121) and appears crucial to maintain a stable contact (131). Vinculin may also be playing important roles in lymphocyte chemotaxis that would merit dedicated studies.

One step further along the integrin–actin connection, the actin crosslinking protein α-actinin is also considered an important component of the “molecular clutch” that establishes new adhesions (132). At the cell leading edge of T lymphocytes, α-actinin interacts physically with the β2 chain of the LFA-1 open conformation and is required for directional migration (55). The sequential cleavage of the β2 cytoplasmic tail by cathepsin X promotes interaction with both α-actinin and talin, thereby modulating LFA-1 affinity toward the high-affinity conformation (56, 57).

Probably as important as the leading edge protrusion, the less studied uropod contraction is a key process during extravasation and tissue migration. It is a site of integrin detachment. ROCK1 (90) and MyoIIA heavy chain (98) drive uropod retraction and favor integrin recycling. LFA-1 detachment is promoted by sharpin binding to the αL chain, which antagonizes the open conformation (112).

### Actin-Motility Receptors Interplay

The expression of chemokine receptors is tightly regulated in T lymphocytes and varies as a function of maturation and functional subset (133). This receptor family belongs to the seven-transmembrane domain protein superfamily called G protein-coupled receptors (GPCRs) and includes 18 genes, divided in CXCR, CCR, XCR, and CXCR subfamilies (134). Upon chemokine binding, chemokine receptors change their conformation, transmitting their signal via the activation of G protein heterotrimers, composed of the α, β, and γ subunits. This results in GTP loading of the Gα subunit. G protein activation stimulates phospholipase Cβ (PLCβ) isoforms, Ser/Thr-kinases, phosphatidylinositol 3-kinase-γ (PI3Kγ), and c-Src-related non-receptor tyrosine kinases (135).

Upon stimulation, most chemokine receptors undergo a desensitization process that includes phosphorylation of their Ser/Thr residues located in the C-terminal region by GPCR kinases (GRKs). This abrogates G protein coupling, promotes binding of β-arrestins, and ultimately induces endocytosis of the chemokine-bound receptor (136). Alternatively, GRKs may phosphorylate ligand-free (non-engaged) chemokine receptors, which also prevents G protein coupling. These processes favor redistribution of receptors to assist T lymphocytes in sensing changes in local concentrations of chemokines (134).

Key signaling proteins integrate the interplay between chemokine receptors, integrins, and cytoskeleton dynamics. Rap GTPases are crucial in promoting adhesion in response to chemokine or antigen stimuli. In T lymphocytes, chemokine stimulation leads to Rap1 activation that binds and activates RapL, which acts as an adaptor and activator of the kinase Mst1. The RapL/Mst1 complex has the ability to bind either to β1 or β2 integrins and induces their conformational activation (106). Furthermore, results obtained with primary T cells show that Rap1 activation favors LFA-1 transport to the leading edge in vesicles also containing the RapL/Mst1 complex (91). In humans, Mst1 deficiency is associated with a severe naïve T cell lymphopenia (92), in agreement with the role of this kinase in thymic egress as observed in the corresponding murine model (93). Rap1 activation is driven by several GEFs, such as CALDAG-GEF1 and the CRKL/C3G complex (107, 108). Homozygous mutations in the gene encoding CalDAG-GEFI have been reported in patients with a form of leukocyte adhesion deficiency (LAD-III). In these patients, T lymphocytes display defects in LFA-1 activation, in particular following CXCL12 stimulation (109). Many actin-related proteins are involved in the activation and transport of these proteins to the membrane. Phospholipase C promotes the delivery of Rap1 vesicles to the leading edge (110) and activates CALDAG-GEF1 through Ca^2+^ and diacylglycerol (DAG) generation (107). On the other hand, WAVE2, Arp2/3, and ABL are required for the transport of the CRKL/C3G complex to the membrane (121).

### Actin–Antigen Receptor Interplay

In addition to LFA-1, the TCR and its associated signalosome require the actin cytoskeleton and remodel it (137). As we will see in this chapter, the main impact of TCR signaling on T lymphocyte motility is the delivery of a stop signal to assemble the immunological synapse. The actin cytoskeleton provides a mechanical platform for TCR signaling at the immunological synapse. The first evidence for this cross-talk came from an early study that used a fractionation approach showed anchorage of cell surface expressed ζ chain of the TCR/CD3 complex to the cytoskeleton in resting T cells (138). Over the last 10 years, it has become clear that the TCR and signaling modules are distributed as microclusters. At the immunological synapse, these microclusters follow centripetal motion driven by a combination of actin flow and actomyosin contraction (52, 139–142).

An important function of TCR signaling is to promote the conformational activation of LFA-1 (143). In this context, how both receptors interact in modulating the remodeling of the actin cytoskeleton was unclear. Using a micropatterning approach, a recent study established that the TCR stimulates actin network extension downstream of LFA-1. This generates the cytoskeletal tensions necessary to mechanical sensing and T cell spreading (144).

The amplitude and quality of an antigen-specific T cell response depends on the scanning behavior, and in particular the sampling time that T cells spend in contact with APC. Recognition of cognate antigens through the TCR transmits a stop signal that leads to reduced motility and prolonged contact with the APC (13). The combination of LFA-1 triggering through ICAM-1 and TCR with MHC molecules presenting cognate antigens is sufficient to arrest T cell motility. An initial wave of [Ca^2+^]i is required for the stop signal (145). In addition, the [Ca^2+^]i signaling pattern regulates the shape and stability of T lymphocyte contacts with APC (146). The stopping of T lymphocytes upon APC encounter is also dependent on myosin II (147). Strikingly, during this phase of arrest, T lymphocytes undergo an extensive reorganization of their membrane, cytoskeleton and even internal organelles. As a result, the interface is segregated into a central cluster of antigen receptors and a peripheral ring of adhesion molecules, building up the so-called immunological synapse (148, 149).

Interestingly, a continuum from the asymmetric leading edge of the migrating lymphocyte to the axisymmetric periphery of the immunological synapse of the APC-engaged lymphocyte has been proposed on the basis of the distribution of key molecules (28). Although apparently very distinct, the structural organizations supporting the motile polarized cell and the cell establishing a synapse could then switch easily. Such mechanism would facilitate the successive encounter of multiple APC. Along these lines, live recording rather than imaging of fixed samples reveals that immunological synapse can be motile. Such synapses, termed kinapse, are characterized by a moving adhesive junction and a leading lamellipodium (150, 151). Therefore, synapses should be seen as dynamic and flexible structures that can accommodate T lymphocyte displacement at the surface of the APC. Indeed, time-lapse total internal reflection fluorescence (TIRF) microscopy has been used to show that TCR signaling can occur while the synaptic contact is undergoing translation (152). In this context, collapse of the leading edge was followed by an inward flow of TCR microclusters toward a region impoverished in filamentous actin, as observed in prototypical concentric synapses (40). A recent study combining intravital two-photon microscopy and migration along micro-channels revealed that the kinapse versus synapse behavior of T cells depends on the affinity of the antigen. Interestingly, stimulation with only high-affinity antigen is able to evoke for full arrest, which is mediated via Arp2/3-dependent actin remodeling (153). The requirement for this pathway in stabilizing the immunological synapse is in agreement with our previous observation that T lymphocytes from Wiskott–Aldrich syndrome patients displayed reduced actin polymerization at the immunological synapse and that these cells were abnormally elongated and motile at the contact with APC (119, 154). Accordingly, a study using lipid bilayers showed that WASP promotes immunological synapse symmetry to counteract PKC-θ-mediated immunological synapse destabilization (118). Together, this identifies the WASP-Arp2/3 pathway as central in setting the kinapse versus synapse behavior.

Chemokines have been proposed to modulate the TCR-driven arrest of T lymphocytes. CCL21 has been shown to increase LFA-1 responsiveness to TCR stop signals (155). T lymphocytes migrating on immobilized CCL21 and CXCL12 cluster LFA-1 at the leading edge, which is then primed for activation via the TCR-driven inside-out signal. On the opposite, chemokines that bind CXCR3 and CCR7 have been shown with Transwell assays to override the TCR-mediated stop signal (156, 157). *In vivo*, chemokines may therefore tune the encounters between T lymphocytes and APC by attracting T lymphocytes, favoring arrest and eventually promoting detachment.

Additionally, different surface receptors can modulate the scanning behavior of T lymphocytes as they encounter APC, by tuning the Stop signal. The co-receptor CTLA4 overrides the TCR-mediated stop signal by increasing T-cell motility, thereby inhibiting T-cell activation (158). Anti-CTLA-4 Abs have been tested to activate T cell immunity under numerous preclinical and clinical settings (159, 160). Another inhibitory receptor expressed at the surface of T lymphocytes, PD-1 can modulate the stop signal. Indeed, in a murine model of diabetes, the blockade of PD-1 or PD-L1 in pancreatic islets abrogated peripheral tolerance by favoring the stop signal, which resulted in prolonged interactions with the APC and enhanced T lymphocyte response (161).

*In vivo*, the effect of TCR signaling on the motility behavior has been well characterized in naïve T lymphocytes encountering antigen presented by dendritic cells in lymph nodes. Three successive steps have been characterized: (1) transient serial encounters, (2) stable contacts leading to full T lymphocyte activation, and (3) high motility and rapid proliferation (24). Therefore, during an immune response, the motility behavior of T lymphocytes is changing dramatically. Interestingly, within the same tissue, the motility behavior of T lymphocytes might be heterogeneous. Time-lapse video recording by two-photon microscopy revealed distinct behaviors, from rapid single-cell migration along random paths to collective behaviors as highly dynamic T cell swarms or stable T cell clusters (23, 162). This latter observation might correspond to the phenomenon of homotypic clustering, which is secondary to the encounter with dendritic cells and results in the formation of T cell-T cell synapses that allow concerted activation via cytokine sharing (163).

## Actin Cytoskeleton Interactions with the Plasma Membrane

The plasma membrane is the interphase between the cytoplasm and the extracellular environment and the physical support of the cell receptors that sense extracellular cues. As such, it is a key element to consider in the context of T lymphocyte motility. There is a compartmentalized interplay between the plasma membrane and the actin cytoskeleton [for a general review, see Ref. (164)]. At the molecular level, this interplay includes lipid components such as phosphoinositides and the RhoGTPases as master controllers of actin-regulatory proteins. In addition, there is a physical interplay via membrane-associated proteins that can anchor the actin cytoskeleton (ERM proteins) or that concomitantly govern actin dynamics and membrane curvature (BAR domain-containing proteins).

### Phosphoinositides

The role of phosphoinositides in the regulation of actin dynamics has been studied in many cell types including T lymphocytes. The triggering of most of the chemokine receptors expressed by T lymphocytes is accompanied by a robust activation of phosphoinositide 3-kinase (PI3K) (102). This leads to the accumulation of PI(3,4,5)P3 at the leading edge (103). In contrast, at the rear of the cell, membrane phosphoinositide levels contribute to cell detachment since the hydrolysis of PI(3,4,5)P3 by PTEN hampers the protrusive activity. Combination of both processes establishes a guidance axis in the cell. More precisely, chemokine receptors and the TCR activate PI3K that catalyzes the phosphoinositide conversion to PI(3,4,5)P3, which allows the activation of the Rac activator DOCK2 at the plasma membrane. PI3K inhibition affects the *in vivo* motility of T lymphocytes with effects on migration speed (103) and directionality (66). DOCK2 deficiency is also affecting T lymphocyte motility, as recently identified in a cohort of immunodeficient patients carrying DOCK2 mutations. T lymphocytes from these patients were found to be defective in actin polymerization and directional motility in response to chemokines (67). Importantly, changes of local concentrations of phosphoinositides may modify the relative prevalence of specific actin structures such as branched or unbranched networks. This can in part be mediated by the propensity of PI(4,5)P2 to inhibit profilin activity (104, 105). How such regulation applies to the context of T lymphocyte motility remains to be explored. The distribution of phosphoinositides is also tightly regulated at the immunological synapse (40), but this aspect is beyond the scope of the present review.

### RhoGTPases

RhoGTPases are master controllers of cell polarity and shape by regulating protrusive and contractile activities downstream a number of receptors. RhoGTPases belong to the Ras superfamily, cycle between inactive GDP-bound and active GTP-bound forms, which activate effectors (165). This cycle is dynamically controlled by two groups of proteins called GTPase Activation Proteins (GAPs) and guanosine exchange factors (GEFs) that respectively promote inactive and active forms (58). A further level of GTPase activity regulation is exerted by guanosine dissociation inhibitors (GDIs), which act by sequestering the RhoGTPases in the cytosol, preventing their interaction with GEFs and effectors. Post-translational modifications such as phosphorylation or palmitoylation fine-tune the activity of these molecules (166).

Rac1 and Rac2, whose functions usually overlap, are considered the main actin polymerization controllers, exerting their role mainly at the leading edge, where they activate WAVE and Arp2/3 to generate protrusions enriched in branched actin. In T lymphocytes, Rac1/Rac2 activation will be mainly under the control of DOCK2 and Vav1 GEFs, both of which are activated under chemokine receptors and the TCR (58).

At the back of the cell, RhoA is described as the key molecule controlling uropod contraction. RhoA activates ROCK, which binds and phosphorylates the Myosin Light Chain, thereby inducing the contraction of actomyosin bundles. By governing uropod detachment, this pathway is central for T cell chemotaxis (111). Although a front-back cell polarity is established through Rac1 and RhoA, RhoA activity is also present at the leading edge, where two of its main effectors are though to be MLCK and mDia1 (90). This might generate an internal polarity of Rho actions depending on the local presence of its effectors.

Cdc42 controls the assembly, collapse and reorientation of cell polarity, which are determinant steps in the chemotaxis of T lymphocytes. This RhoGTPase promotes the generation of filopodia, which sense the environment and prepare the assembly of the lamellipodium. In T lymphocytes, Cdc42 stimulates WASP activity, which promotes Arp2/3-dependent actin branching. Cdc42 is activated downstream of chemokine receptors through different GEFs, including DOCK8, which deficiency causes a combined immunodeficiency (68, 69). DOCK8 appears to activate Cdc42 specifically at the cell leading edge. Defective expression of DOCK8 causes a T cell migration defect in confined spaces, with a phenotype of abnormally elongated cells associated to cell death by fragmentation (70). In addition, by interacting with the polarity complex Par, Cdc42 plays a key role in polarizing several organelles including the Golgi apparatus, the nucleus and the MTOC (106).

A remarkable work has been done in dissection the role of the main RhoGTPases. Still, the promiscuity with which RhoGTPases interact with effectors and regulators, the modulation of their activity depending on their location as well as the existence of yet poorly characterized family members, remain questions to be addressed. In that respect, the use of dedicated biosensors and optogenetic tools appears promising.

### BAR Domain-Containing Proteins

Interplay between plasma membrane and cytoskeleton goes beyond signaling connections. Indeed, there is a concerted regulation of the physical properties of both elements. This is mostly controlled by a heterogeneous group of BAR domain-containing proteins that have the ability to promote and respond to membrane curvature changes (167). In addition, some of those proteins can also recruit and modulate different actin regulators. For example, the protein srGAP2 binds and inhibits the formin FMNL1 (113). The protein CIP4 appears to play the role of a switch between actin elongation and branching activities. Indeed, it activates Arp2/3 complex through interaction with WASP, while inhibiting the formin mDia1 (59). The importance of F-BAR proteins in lymphoid cell migration has been illustrated recently in tumoral lymphocytes, where CIP4 regulates lamellipodia assembly and orientation along chemokine gradients (60). CIP4-deficient T lymphocytes display defective adhesion to VCAM1 and ICAM1 present at the surface of endothelial cells. Consequently, CIP4-deficient T cells display impaired transendothelial migration (61).

### ERM Proteins

The ezrin, radixin, moesin (ERM) family is composed by cytoplasmic proteins with the capability to bind simultaneously to the actin cytoskeleton and to the cytoplasmic tails of different transmembrane proteins such as integrins, antigen receptors and chemokine receptors (168). ERM proteins are key for T lymphocyte migration, as their over-activation or silencing induces defects in β1 integrin-dependent cell adhesion, TCR response, transmigration, and lymph node homing *in vivo* (74–76). ERM proteins switch from a folded to an unfolded active conformation depending mainly on the phosphorylation state of a threonine residue located in their actin-binding domain. ERM protein phosphorylation state is highly controlled in lymphocytes by kinases such as ROCK, GPCR kinase 2, or PKCs (169, 170). Chemokine stimulation induces dephosphorylation of ERM proteins and resorption of surface microvilli (171). ERM proteins are particularly active at the rear of migrating lymphocytes, where they coordinate contraction and retraction of the uropod (172). This polarized activity is coordinated by RhoGTPases since the presence of a constitutive active RhoA induces a hyper-phosphorylation of ERM proteins and protrusion stabilization, whereas the over-activation of Cdc42 or Rac1 evokes a dephosphorylation of ERM proteins and protrusion disappearance (173).

## Concluding Remarks

The actin cytoskeleton provides a fascinating mechanical framework that endows T lymphocytes with an autonomous motility program. Importantly, the many connections between the actin cytoskeleton and the membrane, receptors, and signaling modules allow an integration of environmental cues so that T lymphocytes adapt their motility behavior. Such tunable motility supports the main missions of T lymphocytes: immuno-surveillance (timely recognition of foreign antigens) and immune defense (mounting of adapted immune responses against pathogens).

Beyond the many facets of actin cytoskeleton remodeling reported in this review, key fundamental questions remain open. To fully appreciate the role of specific actin regulators and better characterize the actin networks elaborated in precise cellular locations, we need more detailed analysis of the actin cytoskeleton microarchitecture. For this purpose, novel methods to visualize actin polymerization combined with super-resolution microscopy approaches appear promising (40, 53, 174). An important challenge is that we need to achieve adequate temporal resolution in the imaging of motile lymphocytes, as the characteristic timescales of lymphocyte migration and actin dynamics are very short. For example, actin filament barbed ends have been shown to assemble at a rate of 3,000 subunits/s (2). This implies that an actin filament can reach length scales equivalent to the diameter of a lymphocyte (10 μm) in <2 s. An additional difficulty is to assess very fine regulation of motility due to cell-to-cell asynchrony within a population, for example if one wishes to study relative timing of GTPase activation within protrusions. This requires single-cell analysis. Cell-to-cell asynchrony could then be solved by computational alignment.

We should be able to learn more from cell shape dynamics, in association with membrane curvature and protrusion dynamics. Existing approaches useful to study the evolution of cell shape, such as kymographs (175) or edge velocity maps (176), have not yet been applied to lymphocyte populations migrating over different substrates. It will also be important to better understand which molecular regulation tunes actin-related biophysical properties to generate tensions and forces. Along this line, the mapping of contractile forces using pillar arrays has recently been applied to T lymphocytes (177). The field also needs to develop integrated approaches that can combine molecular and bio-mechanical data together with motility parameters. Along this line, a recently developed MATLAB-based toolset called TIAM allows for integration of data such as area of attachment to the underlying substrate, cell polarity and fluorescence intensity from two fluorescence channels (26).

Finally, the part of the review dedicated to the actin cytoskeleton regulators studied in T lymphocytes reveals that the function of many proteins remains to be characterized in these cells. Screening approaches combined to high-content automated imaging would accelerate our discovery of novel molecules controlling specific aspects of actin cytoskeleton remodeling during T lymphocyte migration. This knowledge could then be exploited to identify targets for the tuning of T lymphocyte activation in the context of infection, auto-immunity and cancer.

## Conflict of Interest Statement

The authors declare that the research was conducted in the absence of any commercial or financial relationships that could be construed as a potential conflict of interest.
