# Supplementary material for: T Lymphocyte Migration: An Action Movie Starring the Actin and Associated Actors
Source: Front Immunol. 2015 Nov 18;6:586. doi: 10.3389/fimmu.2015.00586 (PMC4649030; doi:10.3389/fimmu.2015.00586)
Supplement: Supplementary file 2 [file Data_Sheet_1.DOCX]

**T lymphocyte migration: an action movie**

**starring the actin and associated actors**

**MOVIE 1 I Actin cytoskeleton dynamics during T cell directional migration.** Movie showing a primary CD8^+^ human T cell expressing Dendra2-LifeAct moving along a CXCL12 gradient created in a collagen IV-coated Ibidi µ-Slide Chemotaxis ^2D^. The cell extends dynamic protrusions and moves towards the source of CXCL12 (top). The blue line corresponds to the track of the cell measured by pointing on its centroid at each time point. The cell was recorded over 288 sec with time frames of 12 sec.
